# Early Onset Epilepsy Caused by Low-Grade Epilepsy-Associated Tumors and Focal Meningeal Involvement

**DOI:** 10.3390/brainsci10100752

**Published:** 2020-10-18

**Authors:** Luca De Palma, Chiara Pepi, Alessandro De Benedictis, Nicola Pietrafusa, Angela Mastronuzzi, Antonella Cacchione, Giusy Carfì-Pavia, Camilla Rossi-Espagnet, Francesca Diomedi-Camassei, Sabrina Rossi, Antonio Napolitano, Andrea Carai, Giovanna Stefania Colafati, Daniela Longo, Paolo Curatolo, Federico Vigevano, Carlo Efisio Marras, Nicola Specchio

**Affiliations:** 1 Rare and Complex Epilepsies, Department of Neurological Science, Bambino Gesù Children’s Hospital, IRCCS, Member of European Reference Network EpiCARE, 00165 Rome, Italy; luca.depalma@opbg.net (L.D.P.); chiara.pepi@opbg.net (C.P.); nicola1.pietrafusa@opbg.net (N.P.); giusy.carfipavia@opbg.net (G.C.-P.); 2 Child Neurology and Psychiatry Unit, Systems Medicine Department, Tor Vergata University, 00133 Rome, Italy; curatolo@uniroma2.it; 3 Neurosurgery Unit, Department of Neurological Science, Bambino Gesù Children’s Hospital, IRCCS, 00165 Rome, Italy; alessandro.debenedictis@opbg.net (A.D.B.); andrea.carai@opbg.net (A.C.); carloefisio.marras@opbg.net (C.E.M.); 4 Neuro-Oncology Unit, Department of Onco-Hematology, Cell and Gene Therapy, Bambino Gesù Children’s Hospital, IRCCS, 00165 Rome, Italy; angela.mastronuzzi@opbg.net (A.M.); antonella.cacchione@opbg.net (A.C.); 5 Neuroradiology Unit, Imaging Department, Bambino Gesù Children’s Hospital, IRCCS, 00165 Rome, Italy; mcamilla.rossi@opbg.net (C.R.-E.); gstefania.colafati@opbg.net (G.S.C.); daniela.longo@opbg.net (D.L.); 6 Neuroradiology Unit, NESMOS Department, Sapienza University, 00185 Rome, Italy; 7 Pathology Unit—Department of Laboratories, Bambino Gesù Children’s Hospital, IRCCS, 00165 Rome, Italy; francesca.diomedi@opbg.net (F.D.-C.); sabrina.rossi@opbg.net (S.R.); 8 Medical Physics Department, Bambino Gesù Children’s Hospital, IRCCS, 00165 Rome, Italy; antonio.napolitano@opbg.net; 9 Department of Neurological Science, Bambino Gesù Children’s Hospital, IRCCS, Member of European Reference Network EpiCARE, 00165 Rome, Italy; federico.vigevano@opbg.net

**Keywords:** drug resistant epilepsy, epilepsy, temporal lobe, meningeal neoplasms, neuroepithelial tumors, pediatrics, surgery

## Abstract

*Background*: Low-grade epilepsy-associated neuroepithelial tumors (LEATs) are a frequent etiology in pediatric patients with epilepsy undergoing surgery. *Objective*: To identify differences in clinical and post-surgical follow-up between patients with focal meningeal involvement (MI) and those without MI within our cohort of pediatric patients with LEATs. *Methods*: We retrospectively reviewed all pediatric patients (<18 y) who underwent epilepsy surgery between 2011 and 2017 at our hospital. Cohort inclusion required histological diagnosis of LEATs and post-surgical follow-up of ≥2 y. We subsequently stratified patients according to presence of neuroradiological MI. *Results*: We identified 37 patients: five with MI and 32 without. Half of patients (19) were drug sensitive at surgery; similar between groups. The group with MI differed mainly for age of epilepsy-onset (0.6 vs. 7.0 y) but not for epilepsy duration (0.9 vs. 1.5 y). Post-surgery radiological follow-up (median 4.0 y; IQR 2.8–5.0 y) did not indicate disease progression. Seizure outcome was excellent in both groups, with 34 patients overall being both drug- and seizure-free. *Conclusions*: Our study identified a new subgroup of LEATs with focal MI and excellent post-surgical outcome. Moreover, this highlights the effectiveness of early surgery in pediatric LEATs.

## 1. Introduction

Low-grade epilepsy-associated neuroepithelial tumors (LEATs) are an increasingly recognized cause of focal epilepsy, particularly in young adults and children [1]. The histopathological substrate of these low-grade tumors is mostly of neuronal or mixed neuronal and glial origin. The most common histotypes are glioneuronal tumors (GNTs)—particularly gangliogliomas (GGs) and dysembryoplastic neuroepithelial tumor (DNTs)—which are occasionally associated with focal cortical dysplasia (FCD) [2,3,4]. LEATs are more often detected in the temporal lobe [5]. 

Drug-resistant epilepsy is often the major, if not exclusive, neurological symptom of LEATs [6,7], so achieving seizure-freedom is critical to improving the patient’s quality-of-life. LEATs-related epilepsy usually starts around 12–14 y, unlike with FCD in which epilepsy more frequently starts within the first decade (4–7 y) [8]. Nevertheless, pediatric experience from one center reported that onset of epilepsy associated with LEATs might be within the first 3 y of life [9].

Lesionectomy for neocortical epilepsy or anterior temporal lobectomy (ATL) for mesio-temporal epilepsies are highly effective, standard procedures for LEATs [9,10]; between 80% and 90% of patients with LEATs achieve a long-lasting seizure-freedom [11,12], with shorter epilepsy duration prior to surgery and gross tumor resections being good prognostic factors [9].

Meningeal involvement (MI) may result from dissemination of cancer cells to both the leptomeninges (pia and arachnoid) and cerebrospinal fluid (CSF) compartments [13]. MI has rarely been reported in children, especially in association with LEATs [14,15,16], though it has always been associated with malignant transformation and poor prognosis [13,16]. To date, no report has described an occurrence of MI in patients with LEATs that has had an excellent neurological and oncological outcome.

The aim of this retrospective analysis of our cohort of pediatric patients with LEATs was to identify differences in clinical and post-surgical follow-up between subgroups of patients with MI and those without MI.

## 2. Materials and Methods

### 2.1. Patients Selection

This single-center study was approved by the local ethics committee. The local ethics committee waived the written informed consent for collection of these data from retrospective review of records. At our institution, we follow about 1200 patients with epilepsy each year. Between 2011 and 2017, 158 patients underwent epilepsy surgery; search of the hospital database for pediatric patients (<18 y) who underwent epilepsy surgery during the same period found 131 patients. We compiled a database on clinical, neuroradiological, and neuropathological data from these 131 patients and filtered according to fulfillment of all study inclusion criteria:

Clinical and EEG datasets pre- and postoperative were complete;

Follow-up of at least of 2 y; 

Preoperative and postoperative magnetic resonance imaging (MRI) available for review;

LEATs confirmed by postoperative histopathology.

Patients with an age at surgery higher than 18 y and patients with non-tumoral etiology of epilepsy were excluded from the analysis.

**Figure 1:**
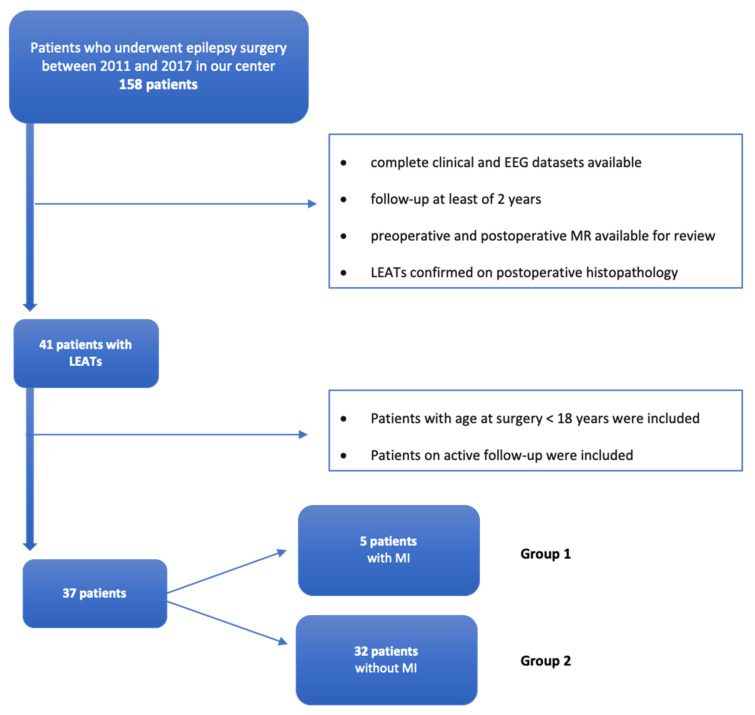
Flowchart illustrating patient selection.

Within our whole cohort of 131 patients who underwent epilepsy surgery before 18 years old, the most common etiologies were (a) LEATs in 37/131 patients (28%), (b) FCD type I and type II in 35/131 patients (27%), and (c) hippocampal sclerosis in 10/131 (8%). Figure 1 shows the flowchart illustrating patients selection.

The study cohort was 37 patients. We subsequently stratified patients according to presence or absence of neuroradiological MI that had been confirmed by histopathology. MRI were acquired on a 3T scanner (Magnetom Skyra, Siemens Erlangen, Erlangen, Germany) and MI was considered positive if meningeal thickening and/or enhancement was observed in post-contrast 3D-T1-weighted sequences (magnetization-prepared rapid acquisition with gradient echo [MPRAGE] or T1-SPACE) [CRE, AN, DL, GSC].

### 2.2. Presurgical Evaluations

All children underwent routine presurgical evaluation. This included full history and neurological examination, video-electroencephalographic recording, and brain MRI. Seizures were classified according to the ILAE Position Paper for Classification and Terminology [17]. Potential surgical candidates were discussed during multi-disciplinary epilepsy surgery meetings to determine suitability for surgery. The decision to offer surgery was based on predicted seizure outcome from presurgical data and surgical risks related to tumor location. All patients underwent neuropsychological evaluation.

### 2.3. Surgical Procedures and Histopathological Diagnosis

The goal of surgery was the removal of the lesion identified on MRI. The surgical procedure included the complete resection of the tumor and adjacent ineloquent tissue up to the next pial border [9]. For lesions involving the mesial-temporal region, a standard anterior-temporal lobe resection was performed. Meninges biopsy was performed in all cases [ADB, AC, CM]. The resected tissue was sent for histopathological examination [FCD, SR].

Histopathologic diagnosis relied on microscopic inspection of surgical brain samples and followed the current 2016 WHO classification [18] and ILAE consensus neuropathological classification system for Focal Cortical Dysplasia and Hippocampal sclerosis [19,20]. In particular, hematoxylin-eosin stain was firstly observed for defining tumor/FCD morphology; further immunostains were performed for highlighting the neuronal and/or glial nature of the tumor. More frequent stains have been: NeuN, synaptophysin, chromogranin and neurofilaments for neuronal histotype; GFAP, EMA, and Olig2 for glial one. In addition, we used CD34 stain for detecting the presence of immature glioneuronal population and Ki-67 for assessing proliferation index. Molecular biology was selectively applied to detect BRAF status, histones mutations, and other tumor-specific genetic alterations.

### 2.4. Follow-Up and Surgical Outcomes

All operated patients were re-evaluated by general examination in the outpatient clinic by the neurosurgeon 2 weeks after surgery. Further follow-up clinical evaluations by multidisciplinary clinicians occurred at 3, 6, and 12 months after surgery and then every 12 months for at least 5 y. Postoperative brain MRI were performed immediately after surgery to exclude potential residual tumor, and subsequently at 6–10-month intervals. The resection was considered complete if the brain lesion was no longer evident from comparison between the preoperative and immediately postsurgical brain MRI. Seizure outcome was recorded using the Engel classification [21]. In seizure-free patients, anti-seizure medications were withdrawn after 3 to 12 months post-operation.

## 3. Results

Thirty-seven patients met the study inclusion criteria, which required histological diagnosis of LEATs, either isolated (28 patients) or in association with FCD type IIIb (9 patients). Part of this clinical series was previously included within aggregated data of two previous reports [22,23]. Demographic data and clinical findings are listed in Table 1 and Table 2 and partly summarized in Table 3. Twenty patients (over half) were males. In all cases, epilepsy was the presenting symptom. Twenty-two patients (about two thirds) experienced focal seizures with impaired awareness and 11 (less than one third) had focal seizures with unimpaired awareness. Evolution to bilateral tonic–clonic seizure was reported in 4 patients. Neuropsychological evaluation revealed that in MI group, one patient out of five (20%) showed a mild intellectual disability (ID) and one (20%) a borderline cognitive level. Among the group without evidence of MI, we found out that three out of 32 (9%) showed a mild ID, one (3%) presented Autism Spectrum Disorder associated with moderate ID, and one (3%) showed a borderline cognitive level. With respect to MI, five patients had LEATs and MI, whereas 32 patients had LEATs without MI.

At surgery, 18 patients (half) were drug resistant according to the current ILAE definition [24]. Long-term video-EEG of at least 12 h was performed in all 37 patients; we recorded habitual seizures in 14 patients (over one third). LEATs were located in the temporal lobe in 23 patients (near two thirds) frontal lobe in seven patients, parietal lobe in six patients, and occipital lobe in one patient.

At the time of this review, the median post-surgical follow-up was 4.0 y (interquartile range (IQR) 2.8–5.0 y), with 34 of 37 patients being both drug- and seizure-free, one patient was seizure-free with ongoing drugs (Engel Id), and two patients still experienced seizures (both of these were drug resistant at surgery). Five patients were re-operated due to incomplete resection and seizure persistence; in three of these five, the previous operation was done in another hospital (Table 4). No patients experienced immediate and late post-surgical complications, and no unexpected post-surgical deficit was evident.

### 3.1. LEATs with MI (Group 1) 

**Figure 2:**
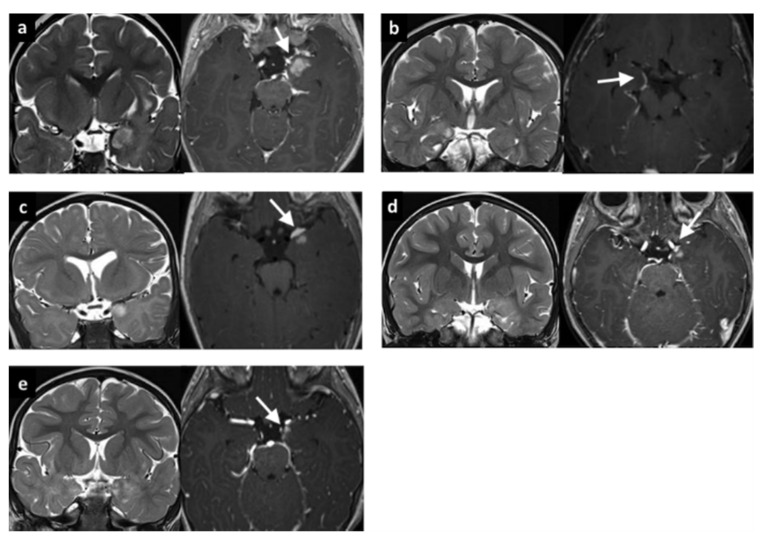
MRI of five patients belonging to Group 1 (age at epilepsy onset <3 y and evidence of meningeal enhancement). All five cases demonstrate clear mesial temporal localization with an enhancement after gadolinium injection along the meningeal layer. (**a**) Patient #1. One-year old girl with a left mesial temporal lobe lesion with nodular parenchymal and meningeal enhancement (arrow). (**b**) Patient #2. One-year, 11-month old boy with a right mesial temporal lobe lesion demonstrating meningeal enhancement along the mesial margins (arrow). (**c**) Patient #3. Ten-month old boy with a left mesial temporal lobe lesion showing anterior nodular parenchymal enhancement with associated meningeal enhancement and thickening (arrow). (**d**) Patient #4. Three-year, 9-month old girl with a left temporal lobe lesion associated with a small nodular parenchymal enhancement and anterior meningeal enhancement (arrow). (**e**) Patient #5. Two-year old boy with a left mesial temporal lesion with partial nodular and meningeal enhancement along the anterior and mesial margins (arrow). MRI, magnetic resonance imaging.

Five patients were included in Group 1 (see Table 1). The median age at epilepsy onset was 0.6 y, with median epilepsy duration of 0.9 y. All patients had a temporo-mesial lesion (1 right-side, 4 left-side) and gadolinium-enhancement was evident with brain MRI in a focal area of the lesion and peripherally along the proximal meningeal layer (Figure 2).

The median age at surgery was 2.0 y. One patient had immediate seizure recurrence after lesionectomy; seizure-freedom was achieved following a second anterior temporal lobectomy. All other patients underwent an anterior temporal lobectomy with resection of the deeper mesial temporal structures, including uncus, amygdala, and hippocampus. For two patients, post-surgical brain MRI revealed the persistence of less than 10% of the lesion.

**Figure 3:**
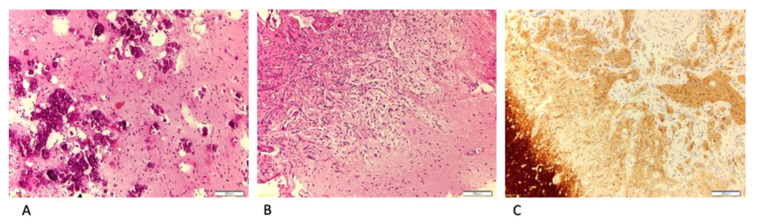
Histopathological findings in patients with LEATs and MI. (**A**) Low-grade tumor with calcification (HE stain, 20×). (**B**) Meningeal infiltration and thickening. We can recognize the nearby cortex in the right inferior quadrant (see arrow) (HE, 20×). (**C**) Evident on the left side is the cortex infiltrated by the tumor and over the center and right side is evidence of MI (synaptophysin). LEATs, low-grade epilepsy-associated neuroepithelial tumors; MI, meningeal involvement.

The median follow-up was 4.1 y (IQR 3.0–4.2 y). At the last radiological follow-up, no patient had modifications of the MI or evidence of diffuse leptomeningeal dissemination (Figure 2). Microscopic examination revealed ganglioglioma in two patients, LEATs not otherwise classified in two patients, and FCD type IIIb (FCD type I associated to low-grade glioma not otherwise classified) in one patient. MRI showed the same tumor infiltration as the intraparenchymal lesion (Figure 3). According to seizure outcome, four patients were classified as Engel class Ia and the other as Engel II.

### 3.2. LEATs without MI (Group 2)

Thirty-two patients were included in Group 2 (see Table 2). The median age at epilepsy onset was 7.0 y, with median epilepsy duration of 1.5 y. Fourteen patients had an extratemporal-lobe localization of the lesion (7 frontal, 6 parietal, and 1 occipital).

The median age at surgery was 9.0 y. Anterior temporal lobectomy was performed in nine patients; the other 23 patients had lesionectomy. Postsurgical brain MRI revealed persistence of less than 10% of operated lesion in five patients; the thalamic part of the lesion was not resected in two patients; and one patient had other lesions (neurofibromatosis) that were not resected.

The median follow-up was 4.0 y (IQR 3.0–5.0 y). Microscopic examination revealed FCD IIIb in eight patients with FCD type I associated to LEATs (4 GG, 2 DNT, 1 astrocytoma, and 1 not otherwise classified); the tumor was isolated in the other 24 patients (9 DNT, 6 GG, 3 glioneuronal tumor, 2 pleomorphic xanthoastrocytoma, 2 low-grade glioma not better specified, 1 pilocytic astrocytoma, and 1 extraventricular neurocytoma). Seizure outcome was classified as Engel class Ia in 30 patients.

### 3.3. Comparison between Group 1 and Group 2 

**Figure 4:**
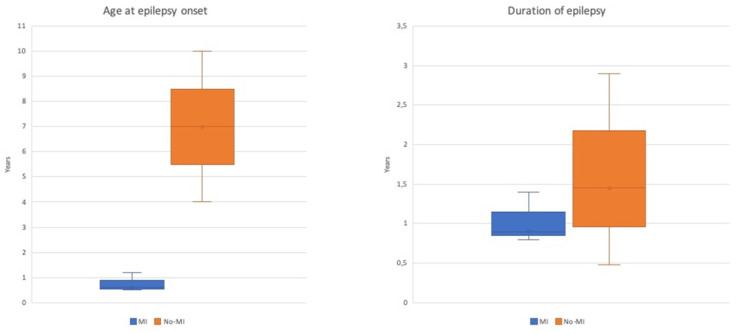
Age at epilepsy onset and duration of epilepsy for the MI (*n* = 5) and no-MI (*n* =3.2) groups. A clear difference in age at epilepsy onset (years) is evident, while duration of epilepsy (years) is similar. Values are median (line), interquartile range (box), and range (bars). MI, meningeal involvement.

Age at seizure onset was the main difference between the groups, being before the first year of life in the main for patients with MI, while generally being above school age (but still within the first decade) for patients without MI. Concordant with the earlier onset of epilepsy, surgery too was at a significantly earlier age for patients with MI; however, duration of epilepsy was similar between the two groups (Table 3 and Figure 4).

The tumor was located exclusively in the temporo-mesial structures for the five patients with MI, while nearly half (14/32) of patients without MI had an extratemporal location for LEATs. The two groups were similar with respect to drug resistance, proportion of complete surgical resection, and seizure outcome (Table 3).

## 4. Discussion

We have described a single center cohort of pediatric patients who were operated for resection of LEATs between 2011 and 2017. Procedurally, all patients underwent the same presurgical clinical, electrophysiological, and brain MRI assessments. Our cohort was limited to patients with follow-up for at least 2 y. Post-surgery, 32 of 37 patients were both drug- and seizure-free, which further supports surgery as an effective treatment for LEATs in the pediatric population [10]. Moreover, the proportion of our cohort that became drug-free (extrapolated to 92%) is larger than has been reported previously (42.9%) [9].

Within our cohort with LEATs, we have described a small homogenous group of five patients (Group 1) with focal MI visible on MRI and confirmed by a neuropathologist. In these patients, brain MRI indicated mesio-temporal LEAT localization and gadolinium enhancement of only the adjacent meninges. Enhancement did not progress in the biannual follow-up MRIs, and we saw no sign of diffuse leptomeningeal spread. The clinical significance of focal MI in patients with LEATs has not yet been described in previous published articles and still remains unclear [15].

Case reports have been sporadic, but some have suggested a secondary non-localized leptomeningeal spread with LEATs [14,15]; however, the spread has been described mainly with malignant transformation or tumoral recurrence with a worse clinical outcome [25,26]. This differs from our patients in whom the MI was present at tumor diagnosis, unassociated with any tumoral progression over the follow-up, and did not influence postsurgical outcome. MI in our patients most likely can be considered as a secondary localization of the original tumor; the MI is probably characterized by the same benign pathogenesis as the intra-parenchymal part, which further histopathological and genetic analysis might confirm.

Compared with the 32 patients without MI (Group 2), the main clinical and radiological differences of Group 1 are an earlier age at seizure onset (within 14 months of birth) and the exclusive localization of lesion within the mesial structures of the temporal lobe. Possibility of an early development of epilepsy in LEATs was already highlighted with other case series [9], but those investigators did not report MI involvement in patients with early age at onset.

Our patient groups were similar in both having a relatively short duration of epilepsy and a high prevalence of pharmaco-sensitivity prior to surgery, suggesting that both groups were evaluated and undertook surgery relatively early in the development of their conditions. In the previously published series, most patients had surgery only after a more prolonged epilepsy duration and after diagnosis of drug-resistant epilepsy [9]. Our study does not established that operating before drug resistance is beneficial; however, this question does warrant further investigation in larger, long-term follow-up studies. Most patients in both groups achieved Class 1a outcome. 

The decision to proceed early with surgery with our patients, even with drug-sensitive epilepsy, considered both the obvious anatomo-electro-clinical association and the absence of functional constraints. The decision for operating specifically prior to drug-resistance was taken mainly due to (a) LEATs-associated epilepsy is characterized by a high prevalence of drug-resistance [5,27]; (b) the rate of malignant transformation is low, but without histopathological and genetic analysis of the tumor specimen, any progression cannot be confidently excluded [7]; (c) the surgery is highly effective (80–90% of Engel 1 at follow-up) with a low risk of complication when eloquent cortex is uneffected [5,11]. 

All our patients who were seizure-free were also drug-free, and early reduction of drugs was not associated with worse seizure outcome when compared with previously reported patients in whom withdrawal of drug was slower [9]. Success with early reduction of drugs has already been highlighted from large cohorts of patients with epilepsy having undergone surgery [28]; hence, we now also support early drug-withdrawal in post-operative patients with LEATs.

Clearly, our two groups comprise radically different numbers, and this heavily affects our confidence in identifying differences, so larger studies would better address our points. Further limitations are related to the retrospective nature of this study. We also do not report any of the recently recognized new low-grade tumoral entities, such as polymorphous low-grade neuroepithelial tumor of the young (PLNTY) and multinodular and vacuolating neuronal tumor (MVNT), which have been described after the end of our review period [29,30].

## 5. Conclusions

All patients had an overall excellent postsurgical outcome in our cohort of pediatric patients who underwent surgery for epilepsy associated with LEATs. Presence of local MI was associated with an early onset of epilepsy but did not change the favorable long-term oncological and epileptological outcomes. Future long-term studies are needed to better clarify the prognosis of patients with LEATs associated with focal MI.

## Figures and Tables

**Table 1:** Clinical and surgical data for patients with low-grade epilepsy-associated neuroepithelial tumors (LEATs) with meningeal involvement (MI) (Group 1).

Case No.	Gender	Age at Onset (y)	Seizure Type	Seizure Frequency	DR	Age at Surgery (y)	Epilepsy Duration (y)	Location	Surgery	Complete Resection	Pathology	Oncological Outcome	F-U (y)	Engel Class	Medication Free	MI
#1	M	0.6	Focal aware, non-motor (behavior arrest)	weekly	yes	2	1.4	R mesial temporal	ATL	Yes	LGG +FCD I	No progression	6.1	II	no	yes
#2	M	1.2	Focal, impaired awareness, tonic	weekly	no	2	0.8	L mesial temporal	ATL	No	LGG	No progression	4.1	Ia	yes	yes
#3	M	0.5	Focal aware, non-motor (behavior arrest)	daily	yes	1.4	0.9	L mesial temporal	ATL	Yes	LGG	No progression	4.2	Ia	yes	yes
#4	F	1.2	Focal aware, non-motor (behavior arrest)	weekly	yes	1.7	0.5	L mesial temporal	ATL	Yes	GG	No progression	3	Ia	yes	yes
#5	F	0.2	Focal aware, motor with automatisms	monthly	no	4	3.8	L mesial temporal	ATL	No	GG	No progression	2.8	Ia	yes	yes

ATL, anterior temporal lobectomy; DR, drug resistance; F, female; FCD, focal cortical dysplasia; F-U, follow up; L, left; LEATs, low-grade epilepsy-associated neuroepithelial tumors; LGG, low-grade glioma; M, male; MI, meningeal involvement; R, right.

**Table 2:** Clinical and surgical data for patients with LEATs without MI (Group 2).

Case No.	Gender	Age at Onset (y)	Seizure Type	Seizure Frequency	DR	Age at Surgery (y)	Epilepsy Duration (y)	Location	Surgery	Complete Resection	Pathology	Oncological Outcome	F-U (y)	Engel Class	Medication Free	MI
#1	F	13	Focal to bilateral	monthly	no	13.9	0.9	L mesial temporal	ATL	Yes	LGG+FCD I	No progression	5	Ia	yes	no
#2	F	9.30	Focal, impaired awareness, non-motor (behavior arrest)	sporadic	no	10.2	0.9	R lateral temporal	Lesionect.	Yes	DNT	No progression	7	Ia	yes	no
#3	M	9.00	Focal aware, non-motor with automatisms	daily	no	9.4	0.4	R mesial temporal	ATL	Yes	GG+FCD I	No progression	6	Ia	yes	no
#4	F	14.0	Focal to bilateral	sporadic	no	14.7	0	L lateral temporal	Lesionect.	Yes	LGG	No progression	5	Ia	yes	no
#5	F	12	Focal to bilateral	daily	yes	13	1	R mesial temporal	ATL	Yes	GG+FCD I	No progression	5	Ia	yes	no
#6	M	6	Focal aware, motor with automatisms	monthly	no	8.9	2.9	R lateral temporal	Lesionect.	Yes	GG+FCD I	No progression	5	Ia	yes	no
#7	F	11	Focal, impaired awareness, non-motor (autonomic)	weekly	no	13	1.9	L mesial temporal	Lesionect.	No	DNT	No progression	4	Ia	yes	no
#8	M	10	Focal, impaired awareness, non-motor (behavior arrest)	daily	no	10	0	L lateral temporal	Lesionect.	Yes	GG+FCD I	No progression	4	Ia	yes	no
#9	F	8	Focal, impaired awareness, non-motor (autonomic)	weekly	yes	11	3	L lateral temporal	Lesionect.	Yes	DNT	No progression	4	Ia	yes	no
#10	F	8	Focal aware, non-motor (autonomic)	daily	yes	9	1	R lateral temporal and thalamic	Lesionect.	No	DNT	No progression	4	Ia	yes	no
#11	F	5.5	Focal impaired awareness, motor with automatisms	weekly	yes	8	2.5	R mesial temporal	ATL	No	GNT	No progression	4	Ia	yes	no
#12	M	5	Focal, impaired awareness, non-motor (behavior arrest)	daily	yes	7, 8	2	L lateral and mesial temporal	ATL	Yes	LGG	No progression	4	Ia	yes	no
#13	F	11.6	Focal aware, non-motor (sensory)	monthly	no	12	0.4	L mesial temporal	Lesionect.	Yes	DNT	No progression	3	Ia	yes	no
#14	M	7	Focal impaired awareness, motor (clonic)	sporadic	no	7	0	L mesial temporal	ATL	Yes	PXA	No progression	5	Ia	yes	no
#15	F	6	Focal aware, non-motor (behavior arrest)	daily	no	9	3	R mesial temporal	ATL	No	DNT+FCD I	No progression	4	Ia	yes	no
#16	M	5.8	Focal impaired awareness, motor (clonic)	daily	yes	5.9	0.1	R parietal	Lesionect.	Yes	GG	No progression	7	Ia	yes	no
#17	M	7	Focal aware, non-motor (behavior arrest)	daily	yes	9	2.0	R parietal	Lesionect.	Yes	PXA	No progression	3	Ia	yes	no
#18	M	8	Focal, impaired awareness, non-motor (behavior arrest)	daily	no	9	1	R parietal	Lesionect.	No	GG	No progression	2	Ia	yes	no
#19	M	9	Focal, impaired awareness, non-motor (behavior arrest)	sporadic	no	9	1	L parietal	Lesionect.	Yes	GG	No progression	2	Ia	yes	no
#20	M	5.1	Focal aware, motor (clonic)	sporadic	yes	8	2.9	R parietal	Lesionect.	Yes	DNT+FCD I	No progression	3	Ia	yes	no
#21	F	4	Focal impaired awareness, motor (clonic)	daily	yes	10	6	R parietal	Lesionect.	Yes	GG	No progression	2	Ia	yes	no
#22	M	14.5	Focal to bilateral	sporadic	yes	15	0.5	L occipital	Lesionect.	Yes	DNT	No progression	3	Ia	yes	no
#23	F	4	Focal impaired awareness, motor with automatisms	weekly	no	4, 10	0	L frontal	Lesionect.	Yes	DNT	No progression	2	Ia	yes	no
#24	M	11	Focal impaired awareness, hypermotor	monthly	no	13.9	2.9	R frontal	Lesionect.	No	Pilocytic astrocytoma	No progression	4	Ia	yes	no
#25	M	10	Focal impaired awareness, hypermotor	monthly	no	10, 16	16	R frontal	Lesionect.	Yes	Extraventricular neurocytoma	No progression	2	Ia	yes	no
#26	F	0.8	Focal impaired awareness, non-motor (behavior arrest)	weekly	no	1	0.4	L mesial temporal	ATL	No	LG Astroc. +FCD I	No progression	8.8	Ia	yes	no
#27	F	1	Focal, impaired awareness, motor with automatisms	daily	yes	3	2	R frontal	Lesionect.	Yes	GG	No progression	5	Ia	yes	no
#28	M	2	Focal impaired awareness, epileptic spasms	monthly	yes	15	13	R frontal	Lesionect.	Yes	GNT	No progression	2	Ia	yes	no
#29	M	3	Focal, impaired awareness, motor with automatisms	weekly	yes	4	8	L mesial temporal	Lesionect.	No	GG	No progression	3	Ia	yes	no
#30	M	3	Focal, aware, non-motor (behavior arrest)	daily	no	15	12	L mesial temporal	ATL	Yes	DNT	No progression	2	Id	no	no
#31	F	2.90	Focal, impaired awareness, clonic	weekly	yes	5	2.1	L frontal	Lesionect.	No	DNT	No progression	2.5	III	no	no
#32	M	3	Focal, impaired awareness, tonic	daily	yes	8	5	L frontal	Lesionect.	Yes	GNT	No progression	3	Ia	yes	no

Patients #12, #23, and #25 had repeated surgeries at the age of 7 and 8 y, 4 and 10 y, 10 and 16 y, respectively. Astroc., astrocytoma; ATL, anterior temporal lobectomy; DR, drug resistance; DNT, dysembryoplastic neuroepithelial tumor; F, female; FCD, focal cortical dysplasia; F-U, follow up; GG, ganglioglioma; GNT, glioneuronal tumor; L, left; LEATs, low-grade epilepsy-associated neuroepithelial tumors; Lesionect., lesionectomy; LGG, low-grade glioma; M, male; MI, meningeal involvement; PXA, pleomorphic xanthoastrocytoma; R, right.

**Table 3:** Descriptive statistics for selected clinical and surgical data of the two groups and cohort.

Variable	Group 1	Group 2	Total
Number (% of total)	5 (13.6%)	32 (86.4%)	37 (100.0%)
Median age at seizure onset (IQR), y	0.6 (0.5–1.2)	7.0 (4.0–10.0)	6.00 (3.0–9.3)
Median age at surgery (IQR), y	2.0 (1.7–2.0)	9.0 (7.7–12.3)	9.0 (5.0–11.0)
Median duration of epilepsy (IQR), y	0.9 (0.8–1.4)	1.5 (0.4–2.9)	1.0 (0.5–2.9)
Tumor location, temporal/extratemporal	5/0	18/14	23/14
Degree of tumor resection, total/partial	3/2	25/7	28/9
Seizure outcome, Class Ia/All other	4/1	30/2	34/3
Drug resistant/responsive	3/2	15/17	18/19

Group 1: LEATs with MI; Group 2: LEATs without MI. IQR, interquartile range; LEATs, low-grade epilepsy-associated neuroepithelial tumors; MI, meningeal involvement.

**Table 4:** Clinical findings in re-operated patients.

Case No.	Age at Onset	Location	Pathology	MI	Complete Resection	Outcome
#1	5 y	L lateral and mesial temporal	LGG	no	yes	Ia
#2	3 y	L mesial temporal	GG	no	yes	Ia
#3	0.5 y	L mesial temporal	LGG	yes	yes	Ia
#4	11	R frontal	Pilocytic astrocytoma	no	yes	Ia
#5	4	L frontal	DNT	no	yes	Ia
